# Effects of Live Music on the Perception of Noise in the SICU/PICU: A Patient, Caregiver, and Medical Staff Environmental Study

**DOI:** 10.3390/ijerph20043499

**Published:** 2023-02-16

**Authors:** Andrew Rossetti, Joanne Loewy, Wen Chang-Lit, Nienke H. van Dokkum, Erik Baumann, Gabrielle Bouissou, John Mondanaro, Todd O’Connor, Gabriela Asch-Ortiz, Hayato Mitaka

**Affiliations:** 1 The Louis Armstrong Center for Music and Medicine, Mount Sinai Health System, Icahn School of Medicine, New York, NY 10003, USA; 2 Independent Researcher, New York, NY 11354, USA; 3 Beatrix Children’s Hospital, 9713 GZ Groningen, The Netherlands; 4 International Association for Music and Medicine, Lima 15074, Peru; 5 NYC Health + Hospitals/Metropolitan, New York, NY 10029, USA; 6 Brookdale Department of Geriatrics and Palliative Medicine, Mount Sinai Hospital, New York, NY 10019, USA; 7 The Mount Sinai Kravis Children’s Hospital, New York, NY 10029, USA; 8 Morgan Stanley Children’s Hospital, New York-Presbyterian, New York, NY 10032, USA; 9 Division of Allergy and Infectious Diseases, Department of Medicine, University of Washington, Seattle, WA 98195, USA

**Keywords:** hospital environments, ICU noise, environmental music therapy, noise perception, music perception, fragile hospital environments

## Abstract

Intensive Care Units (ICUs) require a multidisciplinary team that consists of, but is not limited to, intensivists (clinicians who specialize in critical illness care), pharmacists and nurses, respiratory care therapists, and other medical consultants from a broad range of specialties. The complex and demanding critical care environment provides few opportunities for patients and personal and professional caregivers to evaluate how sound effects them. A growing body of literature attests to noise’s adverse influence on patients’ sleep, and high sound levels are a source of staff stress, as noise is an ubiquitous and noxious stimuli. Vulnerable patients have a low threshold tolerance to audio-induced stress. Despite these indications, peak sound levels often register as high, as can ventilators, and the documented noise levels in hospitals continue to rise. This baseline study, carried out in two hospitals’ Surgical and Pediatric Intensive Care Units, measured the effects of live music on the perception of noise through surveying patients, personal caregivers and staff in randomized conditions of no music, and music as provided by music therapists through our hospital system’s environmental music therapy program.

## 1. Introduction

### 1.1. Background

The Surgical and Pediatric Intensive Care Units (SICU and PICU) require multidisciplinary teams that consist of, but are not limited to, intensivists (clinicians who specialize in critical illness care), pharmacists and nurses, respiratory care therapists, and other medical consultants from a broad range of specialties including surgery, anesthesiology and other specialties. The ideal ICU will have a team representing dozens of different health care professionals and practitioners who assist in patient evaluation and treatment. The intensivists will provide treatment management, diagnosis, interventions, and individualized care for each patient recovering from severe illnesses. This complex matrix of intensive care is accompanied by a number of factors contributing to significant patient and staff stress. Prevalent among these resultantly noxious stimuli is noise generated from multiple sources, and include triple-beat alarms, staff conversations, the drone of noise coming from bedside dialysis machines, and a myriad of other sounds that contribute to a sub-optimal environment for healing [1]. The complex and demanding critical care environment provides few opportunities for caregivers to evaluate how sound impacts patients, and themselves. A growing body of literature attests to noise’s adverse influence on patients’ sleep [2,3,4,5,6] and may contribute to hospital-acquired delirium [7]. Additionally, high sound levels are a source of staff stress [8]. Noise is a ubiquitous and noxious stimulus present in many hospital environments. Research has pointed out that the maximum level of noise at night in hospitals should not exceed 40 decibels (dB) continuous sound level and be no more than 30 dB during the course of the day [9]. Hospital patients often have a low threshold of tolerance to additional stress caused by noise. Despite these indications, studies found that ICU peak sound levels may resister as high as 90 dB [10] to 101 dB [7]. A ventilator itself can generate sound levels of 85 dB [11]. As a comparative orientation to sound level, a motorcycle generates 90 dB, and a vacuum cleaner 70 dB [12]. Despite recommendations, the documented noise levels in hospitals continue to rise.

### 1.2. Environments

Winton Churchill famously stated “We shape our buildings, thereafter they shape us” [13], and this seems especially relevant in examining the environmental factors comprising a SICU experience. The evaluation and design of hospital environments have come to the forefront in recent times. There is growing interest [14,15,16,17] in obtaining less stressful, holistically focused healing spaces. Few would argue that a determining factor of optimal treatment lies in patients’ perception of the hospital experience. Visionary nurse Florence Nightingale recognized that noise was a health hazard in 1859, as evidenced in her observation that “Unnecessary noise is the most cruel abuse which can be inflicted on the sick or the well” [18] (p. 34). While purely architectural elements are often the focus, it is important to acknowledge that environments are made up of unique individual factors that are experienced as an integrated whole, and not a series of isolated events [19,20]. Perceptions of an environment and the resultant reactions to it are prompted by our extended sensory system [21]. The concept of unified sensory perception in the patient/hospital experience is critical, as sound strongly affects and has the potential to modulate the perception of this experience.

### 1.3. Working Definition of Noise

Hilton [22] (p. 284) defined noise “as any sound that is unwanted, undesirable or without musical quality” and coincides with Nightingales’ concept of unwanted sound. Sound and, by extension, noise are complex phenomena. Cultural and social factors, as well as the nature of the sound, and subjective associations of the listener influence how sound is experienced [23]. Most research on hospital soundscapes has focused primarily on sound levels and yet, only a few studies describe the patients’ experience of noise and sounds in the ICUs’ patients’ recovery [24,25,26].

### 1.4. Music in the PICU and SICU

It is well accepted that the inclusion of music in a therapeutic plan of care (particularly live music emphasizing rhythm and collaborative elements of play) can have a constructive impact on the physical, neurologic, psychologic and spiritual domains of the patient experience [27,28,29]. The observation that music can be both orienting and soothing in hospital contexts—constructively re-contextualizing perceptions of environments—has resulted in the increased study of music therapy in a myriad of patient populations. In Critical Care, provided in PICUs and SICUs, the inclusion of recorded music, music with imagery and live music is increasing and under study, though further research is required [30,31,32,33,34]. Some of the impact of music, particularly live music, may specifically help patients in pain or in their feeling out of control and disoriented [32]. The resulting emotional upset may be further exacerbated by disturbances caused by noise levels and unwanted sound in modern intensive care units (ICUs) [35,36,37]. These disturbances along with other causal factors contribute to sleeplessness, which can add to the problem [38,39,40,41]. Patients in the intensive care unit (ICU) exhibit disturbed sleep patterns often attributed to environmental noise [42]. Novaes et al. [43] performed a cross-sectional comparison of factors that affect perceived comfort in the ICU. The greatest stressors were identified as pain, inability to sleep and the discomfort of tubes attached to the nose and mouth. This study points out that there were virtually no differences in the factors contributing to discomfort reported by patients and their families. However, both Novaes et al. [43] and several other studies found that staff perceived the intensity of stressors experienced by patients to be higher than the perception of those experienced by the patients [44,45,46]. In other clinical settings, the incorporation of music into the therapeutic environment also impacted staff, which was shown to indirectly affect treatment outcomes [47]. Moreover, noise control in ICUs has been found to increase staff satisfaction and lower their stress [47]. In such cases, environmental music therapy (EMT) can be an effective means of modulating the staff perception of noise and rendering it as a less noxious stimulus [48].

### 1.5. Pain

Pain is a closely monitored parameter that affects vitality and recovery. Music pre- and post-surgery has been effective for adults [49] and children in reducing pain, serving as an integration or re-focusing intervention during painful procedures such as veni-puncture, [50,51,52] or anticipatory fear [53] and debridement [54]. Richard-Lalonde et al. [31] conducted a systematic evaluation and meta-analysis of 18 randomized controlled trials comparing standard care or noise reduction to determine the effect of music interventions on pain in the adult ICU. It was concluded that a 20–30 min music intervention was effective in reducing pain in adult ICU patients who were able to self-report.

### 1.6. Music Therapy

Music therapy in the treatment of pain has evaluated the impact of music listening, which has largely included the use of recorded music. Studies have measured stress hormones, SIGA and oxytocin. The majority of studies in the medical literature within the past decade have not always assessed the impact of live music. Furthermore, clinical trials conducted amongst patients have not taken into account patient-preference, nor the analysis and characterization of the music used (e.g., the specific music “prescription”) [55]. The varied musical preferences of patients include integral parameters that define music characterization, and attend to the significant area of personal and geographical culture. Culture has been identified as a significant domain of critical care, as reflected by JACHO [56].

In a study of the implementation of live music on agitation in the ICU, patients in the music group did not require any additional sedation with propofol, whereas controls sometimes required propofol to allow sufficient patient–ventilator coordination [57] All patients in the music group were designated grade 2 on the Ramsey scale (a nursing index of need for sedation), whereas patients in the non-music group received a score of 1, (suggesting inadequate sedation—chi-square test, *p* < 0.05.) The effect of music intervention also seemed to last beyond the period during which recorded music was played. Significant differences were found between music and controls in levels of dehydroepiandrosterone, growth hormone, IL-6, and epinephrine (hormonal measures of illness-related stress). Serum levels of dehydroepiandrosterone rose significantly in the controls, while active arm participants maintained more desirable lower levels.

Music therapy is an increasingly recognized intervention in addressing the needs of patients who are approaching the end of life. Music’s transient qualities have served patients in sleep and sedation [50,58,59,60], in assisted relaxation in preoperative anxiety [53] and post-surgery [61,62]. Music therapy has assisted a host of clinical issues in hospital care. A problematic factor presented in the literature is that the majority of studies implement recorded music listening experiences. Although patients might prefer listening to playing, when recorded music is used, there is no flexibility for the therapist to “entrain” (calibrate the music’s periodicity) to the vital signs. This lack of methodological flexibility could engender a host of clinical problems through unwanted stimulation; such as an undesirable fluctuation/increase in heart rate or respiratory rate, for instance, if a recording is increasing in speed or intensity. Having a trained music therapist implement a patient’s live music of choice, through assessing clinical criteria and recognition of the interplay of the music’s characteristics can create stability and a feeling of empathic listening. In addition, if the patient wishes to engage in music-making, the therapist can monitor the desirable effect and outcome in a moment-to-moment dialogue. If a patient chooses to listen, the therapist can entrain the live music to the pulse (heart rate) and/or breathing rate of the patient. In this way, the music experience offered is safer and more sensitively instituted.

Studies addressing music therapy in hospital contexts have shown it to be effective in various stages of care, with the vast majority of studies incorporating “relaxing” music and specific protocols to address symptom management [63,64] While pain and the perception of disease are important parameters that have a direct impact on coping and comfort, so too are one’s personal resources and resilience that enhance their capacity to withstand procedures and various interventions that are necessary components in combating illness. Music therapy can foster resilience [59], though more research is required.

### 1.7. Environmental Music Therapy

EMT was first defined by Joanne Loewy and initiated in the NICU at Beth Israel Medical Center more than two decades ago [48]. Environmental music therapy (EMT) is the intentional use of live music and sound to modulate the soundscape of an area or space. EMT is a developing modality that finds its fundament and takes its cue from the growing interest in the evaluation and design of hospital environments to obtain less stressful, holistically focused healing spaces. It addresses the centrally important issue of the control of hospital sound, and psychoacoustics has focused on the frequency of noise and its decibel levels, when they are in reality a fraction of the issue. The composite sound in an environment that makes up its soundscape supplies meaning about that environment to its constituents through its syntax and semantics, impact, and the associations and metaphors it evokes. A key to changing the perception of a sometimes oppressively perceived hospital soundscape lies in the sensitive and methodical application of EMT to incorporate noise into a newly created “soundtrack” that can have the potential to modify the perception of hospital environments in real time, to its greatest stakeholders; patients, caregivers and staff. A current definition is: “A trauma-informed intervention that uses the metaphoric and associative properties of live music in a dynamic process relying on attunement to an environment and entrainment to its constituents to provide a soundtrack for that environment that modulates the constituents’ perception of the meaning of that space and its soundscape” [48] (p. 131).

### 1.8. Current Study

Music therapists are trained to work with sound, and may sensitively influence the way sound is organized in stressful environments through mechanisms such as entrainment and modulation of discreet music elements [48]. Music and other sounds both include elements such as timbre, pitch, volume, complexity, and intensity, among others. These common elements permit the integration of music with other sounds (such as noise), which is the basis for EMT. Unwanted sound, in the form of monitor alarms and other mechanically generated noise, the sounds of the ill, and staff-generated sound may confound the flow, order, and at times the impact of collaboration, which is instrumental to the care rendered in an intensive care unit, can be perceived by patients, their caregivers, and staff alike as a hostile environment [65].

In addressing the sensitivity and response to unwanted sound through learning about the perceptions of an environment’s constituents, clinicians, patients, families and caregivers might attend to how music may or may not create order and favorably modulate the perception of the environment through subtle adjustments, which include alteration of perception and the meaning of unwanted sound. This process is based on principles of integration of noise into a musically altered soundscape and the orders of musical structure and its effects at any given moment in the ICU [55].

Infusion of a natural, subtle and aesthetically pleasing intervention of music does not require attention or invasive implementation on any person’s part. Music is an organic stimulus that can gradually and sensitively increase awareness and shift one’s state of attention. The outcomes of such an intervention could have serious effects on not only the noise level and perception of its meaning, but also on the perception of the healing environment itself amidst myriad potentially traumatic situations. Since the implemented culture of safety and warnings to its lack thereof are cited as among the highest risks factors that potentially lead to medical errors in America, adherence to measures that can potentially impact the sound environment are well warranted [66]. It is hoped that the current study will enhance attentiveness to sound and perhaps lead to the recognition of interventions, such as environmental music therapy, which may in the future have an influence on the potential means of how music can attend to the level and perception of noise, as well as coherence, that can be achieved in an ICU environment.

## 2. Methodology

The study aimed to investigate the effects of noise perception and the impact of environmental music therapy on 75 patients, 75 caregivers and 72 staff members via a questionnaire and interview. The patient criteria included the following groups: Cancer, Spine, General Surgery and Gynecology, and was adjusted for the severity of illness with a widely used severity index (APACHE II), meaning that patients were included in one of three groups: low, med, and high severity, graded for inclusion; with 25 per group. Staff participation was projected to include: 4 ICU Attending Physicians, 26 Surgery residents, 10 Consultant Attending Physicians, 20 Nurses, 6 Respiratory Therapists, and 6 Physician Assistants. We did not add a control group to this study as we devised a baseline research project that sought to identify what the literature has already characterized as a “hazard”; in particular, “environmental noise”. This study set out to further explicate how sound and noise are perceived by patients, caregivers and staff, and furthermore to assess and evaluate if and how music might change these perceptions in addition to attending to the actual noise on the SICU.

**Figure 1:**
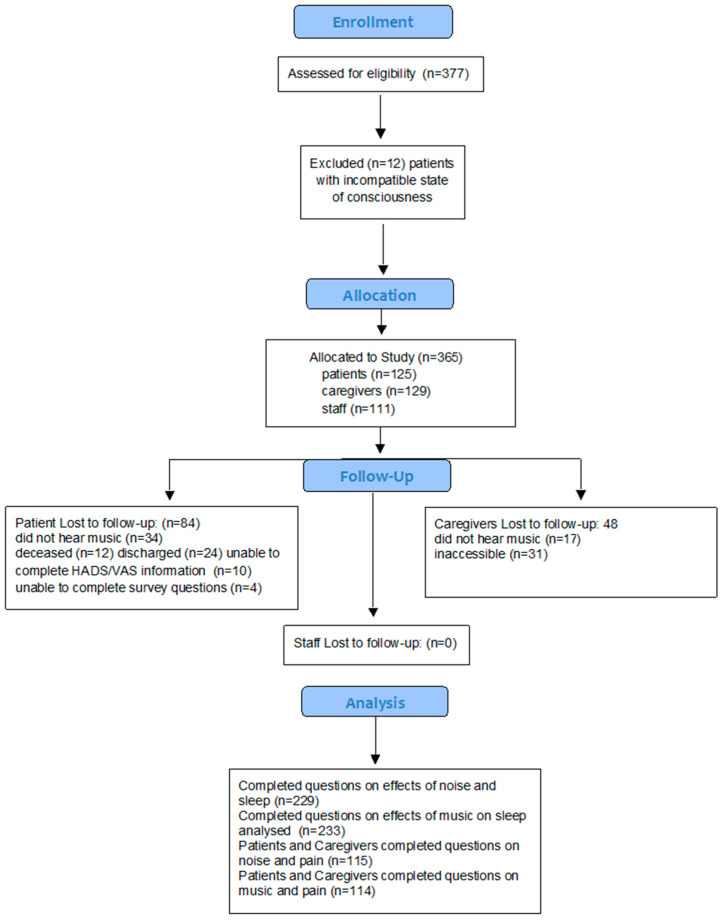
Consort flowchart of participant accrual and flow through the trial.

The impact of noise on the perception of the sound environment determined by a survey for each of the groups mentioned above occurred during the initial enrollment period. The staff participants were invited by the Director of the SICU to participate and were surveyed within one to three months. Follow-up for staff included a re-evaluation during month five and six. Staff participation in the study consisted of the initial enrollment and re-evaluation survey questionnaires as well as active listening while EMT was provided to patients on the SICU. Surveys for patients and caregivers were conducted based on length of stay (LOS) and included questionnaires for both patients and caregivers during their first 24 h period; and a follow-up during their last 24 h period. In general, we tried to receive enrollment for patients and caregivers within their first 24 h of admission to the SICU or PICU as a control (no music) condition and then immediately after the post-condition (music). Staff, on the other hand, received these conditions at varying points. In cases where patients were based in the SICU for less than 24 h, every attempt was made to survey within the first few and final few hours of habitation in the SICU. Patients included those not on ventilators, with a Glasgow Coma Scale of 15 (fully alert) (See Figure 1). The amount of analgesia (morphine, fentanyl, dilaudid, Percocet, oxycodone and methadone) as well as sedatives (versed, propofol, Xanax, haloperidol or Haldol) was determined via the Braden sedation scale. The patients enrolled had the capacity to consent. Questionnaires were gathered in the morning and afternoon. The impact of the sound environment was surveyed twice per day with music or no intervention based on randomization of assignment.

With consideration of the past 5 years’ census prior to the study start, the Physician Director of the SICU determined that 75 patients, 75 caregivers and 72 staff members was a viable number of patients, caregivers and staff to allow for sufficient data collection within a one- to three-year period. The primary investigator of the study met with the statistician who would be analyzing the data and who surmised what the outcome determinants would be for analyzing significance or otherwise.

EMT based on the pilot studies carried out by Stewart and Schneider [67], Schneider [68], Chestnut et al. [69], and Mahoney and Zhang [70], and informed by Rossetti’s recent paper [48], was provided in the intensive care environment. The music therapists offered a 30 min EMT intervention upon enrollment of the patient, either in the morning or afternoon. The total number of sessions per patient was 1–3. The number of sessions was indicated by nursing/physician referral in accordance with the length of the patient’s stay on the SICU. EMT involved the music therapists playing instruments, including the singing and playing of favorite genres and pieces or songs as requested by caregivers, staff, or the patients, as well as improvised music that reflected the level of activation, and other discernable psychosocial parameters in the SICU environment. Active participation for study subjects during the EMT session required only focused listening to the musical intervention provided by the music therapists. The music followed a format that allowed for the varying skill sets of the music therapists involved and in tandem with the observed needs of the participants. The guidelines are described in Rossetti’s recent paper on EMT practice [48]. At the end of three weeks (for staff) or upon discharge of the patients and caregivers, the subjects completed post surveys.

### 2.1. Patient and Caregiver Population

Patient subjects were drawn from the population of the Surgical Intensive Care Unit at Beth Israel and the Mount Sinai Kravis Children’s Hospital, both located in Manhattan, New York, US. Patient criteria included the following groups: Pediatric ICU, Cancer, Spine, General Surgery and Gynecology, and were adjusted for the severity of illness with a widely used severity index (APACHE II.) Referrals from doctors, nurses, and social workers of patients who were defined as high acuity (see Appendix A), and who met the inclusion criteria were enrolled. Staff subjects included: ICU Attending Doctors, Surgery Residents, Consultant Attending Doctors, Nurses, and Physician Assistants. Subjects included patients, their family members and/or caregivers, and their staff nurses, doctors and therapists who met the study criteria as identified from the PICU and SICU teams. Caregiver/family member subjects were identified by patients and their medical staff. All subjects were considered regardless of gender or racial/ethnic background except for any subjects that declined to participate in the study.

### 2.2. Staff Population

Staff was invited to participate by the Director of the SICU and the Director of Music Therapy (co-investigators) verbally, and their participation was voluntary and confirmed by signed consent. All staff subjects were considered regardless of gender or racial/ethnic background except for any subjects who declined to participate in the study. Not listed in the data were the questions that helped define what music might be relevant or significant music. These questions included patients’/caregivers’ recent travel destinations, special events, preferred modalities and genres, music history and background (including what instruments they played, if they sing, etc.). In order to assure that staff participation was voluntary and confidential, the enrollment of the staff population included a Waiver of Documentation of Consent and a Waiver of Research Authorization. The consent form and research authorization were given to the staff to read for informational purposes only and returned to the study staff, without identifiers, and demonstrated the consent of staff who decide to participate in the study.

### 2.3. Statistical Methods

Descriptive statistics were used to show participant characteristics. All data were checked for normality using Q-Q plots and Shapiro–Wilk testing. Given that the data were not normally distributed, non-parametric descriptive statistics (i.e., median values and interquartile ranges) and tests were used throughout the study. Patients’ pre- and post-Apache Scores, VAS (visual analogue scale) scores and HADS (hospital anxiety and depression scale) were compared using Wilcoxon-signed tests. Regarding the section on sleep, we calculated the average values of the three questions from both patient and caregiver questionnaires examining the effect of ambient noise on rest, sleep, and fatigue. In the section on pain, we calculated the average values for two questions: one regarding major procedures and the other minor procedures experienced by patients and observed by caregivers. Noise and music conditions were compared using Mann–Whitney-U tests or a Kruskal–Wallis test where appropriate. Effect sizes according to Cohen’s delta were calculated and interpreted as follows: 0.2 was considered small, 0.5 medium and 0.8 large.

### 2.4. Results

In total, 41 patients (17.6%), 81 caregivers (34.8%) and 111 staff members (47.6%) completed the questionnaires on the effects of noise and music in the ICU setting. Staff members were a mix of medical professionals and support staff (See Table 1).

Patients’ Apache scores, HADS scores and visual analogue scale scores for pain at the two time-points of administering the questionnaires were examined. Apache scores were available for adult patients. Median values (interquartile range) were 11 (7) pre-, and 8 (6) post-intervention, which were significantly lower when the music questionnaire was completed compared to the noise questionnaire (*p* < 0.001). The visual analogue scale was also statistically significantly lower when the music questionnaire was completed compared to the noise questionnaire (2 (1.5) pre and 1 (2) post, *p* = 0.022). The HADS anxiety scores were 7 (6) pre and 7 (9) post, and depression scores were 5 (4) pre and 6 (6) post, (*p* = 0.408 and *p* = 0.410, respectively).

### 2.5. Effects of Noise and Music on Sleep and Pain

Overall, 229 patients, caregivers and staff members completed questions on the effects of noise, and 233 on the effects of music on sleep. We found a median value of 5.5 (interquartile range = 4.5) for the effect of noise, whereas the effect of music’s median value was 7.0 (interquartile range = 3.5). This indicated that the entire cohort rated the effect of music on sleep as greater than the effect of noise on sleep, *U* (N_noise_ = 229, N_music_ = 233) = 21,378, *z* = −3.71, *p* < 0.001. When specifically looking at patients and staff members, this pattern was similar, albeit only statistically significant for staff members (*p* < 0.001). For caregivers, the median value for music was lower than the median for noise, albeit not statistically significant (See Table 2).

In comparing the effects between the three groups, we found median values (interquartile range) for the effect of noise on sleep to be 4.33 (5.04) for patients, 5.50 (5.50) for caregivers and 6.00 (4.00) for staff members (Table 2). A Kruskal–Wallis test showed that there were no significant differences between these three groups (H (2) = 4.02, *p* = 0.13). The median value (interquartile range) for the effect of music on sleep was 6.00 (5.17) for patients, 5.00 (7.00) for caregivers and 7.00 (2.00) for staff members. A Kruskal–Wallis test showed that there were differences between these three groups, (H (2) = 23.34, *p* ≤ 0.001). Post hoc Mann–Whitney-U tests using a Bonferroni-adjusted alpha level of 0.017 (0.05/3) were used to compare all pairs of groups. The difference between patients and staff members was statistically significant, *U*(N_patients_ = 41, N_staff_ = 111) = 1433, *z* = −3.53, *p* <0.001, as was the difference between caregivers and staff members, *U*(N_caregivers_ = 81, N_staff_ = 111) = 2878, *z* = −4.29, *p* < 0.001. The comparison between patients and caregivers was not statistically significant (*p* = 0.42). Effect size was large for staff members, but smaller for other groups (See Table 2).

Overall, 115 patients and caregivers completed the questions on the effects of noise and 114 on the effects of music on pain. The effect of noise median value was 3.0 (interquartile range = 5.0), whereas the effect of music median value was 4.7 (interquartile range = 3.5) (See Table 2). This indicated that the total group rated the effect of music on sleep as higher than the effect of noise on sleep, *U*(N_noise_ = 115, N_music_ = 114) = 5516, *z* = −2.09, *p* =0.037. This pattern was also evident when examining patients and caregivers separately, albeit only statistically significant for caregivers (*p* = 0.022). Comparing the effects between patients and caregivers, we found that the median (interquartile range) for the effect of noise on pain experience was 3.33 (4.85) for patients and 3.00 (5.00) for caregivers. Mann–Whitney-U test revealed no significant differences between the two groups (*p* = 0.41). The median (interquartile range) for the effect of music on pain experience was 4.67 (3.80) for patients and 5.00 (3.25) for caregivers. Mann–Whitney-U test revealed no significant differences between the two groups (*p* = 0.43). Compared with sleep, the effect of both noise and music on pain perception, were less pronounced. Effect sizes were small for all groups (See Table 2).

## 3. Discussion: Music’s Effect

While the question of the influence of noise and unwanted sound on patients and staff in hospitals is receiving growing attention in the search for environments that are conducive to healing and well-being, there are presently few studies examining the effects of environmental music therapy [55,67,68,70,71]. Stewart and Schneider’s [67] NICU study provided recommendations for how live music can “organize, frame, and contain” [67] (p. 90) an often unpleasant soundscape. There are virtually no other identified published music therapy studies examining this phenomenon in high-risk stressful hospital areas. This warrants further analysis of the effects of the music that was implemented over the course of the study. This study is the first of its kind in that it examines a varied cohort including patient caregivers and hospital staff’s perception and of environmental music therapy interventions and noise’s impact on pain and sleep. There is an important gap in the knowledge of the effects of music on pain in ICU patients who are known to experience pain, and more so on their caregivers’ perception of its efficacy on their loved ones [31]. More over, studies of music’s impact in ICUs often focus on patient on ventilators or those unable to self-report [72,73,73] or, as in one recent systematic review of 10 music interventions, on using only data collected from a single music exposure [31]. Additionally, while the deleterious effects of environmental noise on the SICU/PICU are well-known and documented, the use of music and music therapy on the perception of sleep is less understood and is understudied, meriting future study. Future research on live music therapy interventions’ efficacy in addressing pain and providing an environment conductive to enhanced sleep patterns as compared to recorded music experiences is warranted.

A prime focus of the current study was to ascertain whether or not participants were able to spontaneously differentiate and recognize the presence of noise in the units and its possible effects on their state and other conditions, and also to contrast that with the identification and possible response to music in that same environment. We made no assumptions that music would improve the environment. This pilot study does not just examine music, but measures the impact of patient preferred music provided by a music therapist in contrast with the usual and customary sounds in busy inner-city ICUs of a hospital system.

An important first step in analyzing how sound interventions are established is an effective strategy for constructively modulating perception through addressing hospital soundscapes. To that end, our study focused on whether the patients, caregivers and staff were actively distinguishing discreet elements of the sound environment, and whether they were, on the one hand, aware of and able to differentiate patient preferred music vs. noise and/or unwanted sounds in the SICU and PICU, and secondly, to what degree those acoustic elements influenced sleep and the experience of pain. We also measured music and noise’s effect on Apache scores, and anxiety/depression on the HADS. We found a statistically significant reduction in Apache scores in the music condition, which may suggest an overall change in state and observable patient signs with exposure to music, though further rigorous study is needed to confirm the outcomes. Interestingly, the data also showed that there was a statistically significant change (*p* = 0.022) in patients’ perception of pain intensity, which can be seen as reinforcing the Apache findings.

While all three groups found a moderate negative influence of noise on sleep, with staff members’ belief that noise had a higher adverse effect on patients’ sleep and complaints about noise on the unit than patients and caregivers, there was a greater diversity in outcomes regarding environmental music therapy’s effect. The median values show that all three groups believed that music had a strong positive effect on sleep, perception of general quality of care, and comfort. There was a statistical significance between the comparisons of each group’s perception of music’s benefits, with staff holding the highest valuation, followed by patients, and lastly caregivers. Patients and caregivers’ responses showed EMT had a greater positive effect on sleep compared to no music and/or noise on mediating pain. The effect of both noise and music on pain perception was less than their effect on sleep. Music therapists’ inquiry into patients’ preferred music styles found them identifying 26 separate genres, with 90% requesting more than one genre, and 10% one individual genre. The most requested style was “Classical”, with 15.2% of patients identifying it as their preferred genre for the interventions, followed by “Jazz” at 13.39%. A number of culturally diverse styles were requested, attesting to the diversity of the patient cohort (Table 3). We chose to specify use of patient-preferred music to assess the feasibility of attempting to provide personal material in this kind of fragile group context. The study suggests this to be a viable approach to creating music soundscapes for the SICU. A limitation is that we were not able to collect participants’ musical backgrounds or exposure to music experiences. Future efforts may be warranted to explore their importance regarding the response to music interventions. We also were not able to explore in-depth the possible differences in response between patient preferred and arbitrarily chosen genre-based music selections. Having encountered data that suggest possible benefits both in noise perception and its ramifications on patients’ evaluation of sleep and pain experiences, we feel that further studies, with an expanded scope that exceeds the limitations of this pilot study, are necessary. Further exploration of the possible direct benefits of live music interventions that ICUs may encounter may add confirmation of music therapy as a viable and highly cost-effective ICU intervention, contributing to an enhanced quality of life for the units’ patients, caregivers, and staff.

## 4. Conclusions

This is the first study to examine the perception of environmental music therapy and noise in a hospital SICU. It is a baseline study of EMT interventions using biopsychosocial constructs that were effective in showing constituents’ awareness of the SICU sound environment in general and specifically the influence of both noise and music on perception. The patients, caregivers, and staff were all attuned to music and noise present in the SICU/PICU soundscape. All three participant groups found music therapy in the hospital environment to be beneficial, and reflected that music had more of a positive impact on sleep and on pain perception compared with noise. The study may serve as an initial point providing a base for a further detailed study of EMT’s direct effects on a myriad of deleterious issues faced by both staff and visitors to the SICU/PICU. The data also reinforce the concept of noise as a noxious stimulus present in hospital environments. It found moderate positive responses in anxiety and depression through listening to patient preferred music. The study points to patients perceiving environmental music therapy to be a positive influence on their perception of sleep, and that both their caregivers and staff perceive it as a positive influence on patients’ sleep as well. As such, it provides a preliminary insight into EMT being a viable strategy to positively influence pain perception, and in as much, will provide a meaningful entry point for defining the most effective way patient preferred music used in EMT interventions can be implemented, though future studies are required with specific interventions designed to address anxiety and depression in the SICU/PICU. A limitation of the study was the difficulty in accruing the initial number of patients due to a greater number of patients not meeting the exclusion characteristics. Future research might follow to guide and support practices that are specific to environmental music therapy in intensive care units.

## Figures and Tables

**Table 1:** Participant characteristics.

Entire Cohort (N = 233)			
Participant Characteristics			
	Participant Age	Participant Gender	
Patient Median (Range)	58 (14–89)	Male 80 (34.3%)	Female 153 (65.7%)
	PT Age Groups+ %	STF Age Groups+ %	CG Age Groups+ %
14–25 y	5 (12.19%)	4 (3.6%)	7 (8.64%)
26–48 y	9 (21.95%)	64 (57.65%)	17 (20.98%)
49–58 y	7 (17.1%)	37 (33.3%)	47 (58%)
59–64 y	7 (17.1%)	6 (5.4%)	10 (12.34%)
65–89 y	13 (31.7%)	0 (0%)	0 (0%)
Patient Diagnosis			
Oncology n = 9 Respiratory n =9 Hepatic n = 2 Circulatory n= 3	Neurological n = 4 Gastrointestinal n = 10	Surgical complications n = 2 Infectious n = 2	
Patient place of origin	NYC Area 27	Extended US 10	Outside US 4
Staff Characteristics	number n (%)	Staff Gender	
		Male	Female
Physicians	n = 28 (25.2%)	21 (81.1%)	9 (18.9%)
Nurses	n = 57 (51.4%)	8 (14.03%)	49 (85.96%)
Nurse Managers	n = 2 (1.8%)	0 (0%)	2 (100%)
Physician Assistants	n = 3 (2.7%)	2 (66.6%)	1 (33.3%)
Support Staff	n = 17 (15.3%)	6 (35.3%)	11 (64.7%)
Students	n = 4 (3.6%)	1 (25%)	3 (75%)

Abbreviations: PT = Patient, CG = Caregiver, STF = Staff.

**Table 2:** The effect of noise and music on sleep and pain in an ICU setting according to patients, caregivers, and staff members.

Sleep				
	No Music: Median (Interquartile Range)	Music: Median (Interquartile Range)	*p*-Value	Effect Size
Total	5.5 (4.5)	7.0 (3.5)	<0.001	0.25
Patients	4.3 (5.0)	6.0 (5.2)	0.28	0.24
Caregivers	5.0 (5.5)	5.0 (7.0)	0.93	0.03
Staff members	6.0 (4.0)	7.0 (2.0)	<0.001	0.76
Pain				
	No Music: Median (Interquartile range)	Music: Median (Interquartile range)	*p*-value	Effect size
Total	3.0 (5.0)	4.7 (3.5)	0.037	0.05
Patients	3.3 (4.9)	4.7 (3.8)	0.70	0.06
Caregivers	3.0 (5.0)	5.0 (3.3)	0.022	0.35

*p*-values are shown of Mann–Whitney-U tests. For subgroup analyses, *p*-values should be interpreted using a Bonferroni correction. Effect sizes are calculated using Cohen’s delta, where an effect size of 0.2 is considered small, 0.5 medium and 0.8 large.

**Table 3:** Patient preferred music genres.

Entries n = 112 Patients Identifying 1 Genre n = 4 (9.75%) Patient Preferred Music Genre n = 41 Patients Identifying Multiple Genres as Favorite Music n = 37 (90.25%)					
Classic rock	n = 9	(8.03%)	Opera	n = 2	(1.78%)
Blues	n= 3	(2.68%)	40′s big band	n = 2	(1.78%)
Broadway	n = 2	(1.78%)	Chinese pop	n = 1	(1.12%)
Folk	n = 4	(3.5%)	Asian Classical	n = 2	(1.78%)
Country	n = 3	(2.68%)	Kletzmer	n = 1	(1.12%)
Soca	n = 1	(1.12%)	Gospel	n = 5	(4.46%)
Salsa	n = 5	(4.46%)	Contemporary Devotional	n = 5	(4.46%)
Latin	n =4	(3.5%)	Reggae	n = 5	(4.46%)
R&B	n = 3	(3.5%)	New Age	n = 8	(7.14%)
Jazz	n= 15	(13.39%)	Classical	n = 17	(15.2%)
Freestyle	n = 1	(1.12%)	Freestyle	n = 1	(1.12%)
Classic pop	n = 7	(6.25%)	Current Pop	n = 4	(3.5%)
Rap	n = 1	(1.12%)	Hip Hop	n = 1	(1.12%)
House	n = 1	(1.12%)			

## Data Availability

Data available on request due to restrictions of privacy. The data presented in this study are available on request from the corresponding author. The data are not publicly available due to HIPPA restrictions.

## Appendix A

**Table A1:** Sample questions from the patient, caregiver, and staff questionnaire on fatigue, sleep, and noise.

PATIENTS	CAREGIVERS
Does noise in the SICU interfere with rest? 1–10	Question 1: Does noise in the SICU interfere with rest? 1–10
Does noise in the SICU interfere with sleep? 1–10	Question 2: Does noise in the SICU interfere with sleep?
Does noise interfere with family and other support interactions? 1–10	Question 3: Does noise interfere with family interactions and other supportive interactions? 1–10
Does the noise in the unit impact your impression of the care you receive? 1–10	Question 4: Does the noise in the unit impact your impression of the care the patient receives? of the general care? of the medical care?
Does the noise in the unit impact your impression of the general care? 1–10	
Does the noise in the unit impact your impression of the medical care? 1–10	
Who is the least considerate of the impact of voice noise on you? Open text	Question 5: Who is the least considerate of the impact of voice noise on you? Open text
Are the staff responsive to your requests to try and decrease noise? 1–10	Question 6: Are staff responsive to your requests to try and decrease noise? 1–10
Does the loudness of the noise affect your perception of staff? 1–10	Question 7: Does the loudness of the noise affect your perception of staff? 1–10
How bothered are you by the sound of the vents? 1–10	Question 8: How bothered are you by the sound of the Vents, IVACS, Monitor Alarms, Voice Noise, Adjacent Patient Noise (Voice, Music or TV), Sound of Procedure Noise? 1–10
How bothered are you by the sound of the IVACS? 1–10	
How bothered are you by the sound of the Monitor alarms? 1–10	
How bothered are you by the sound of Voice noise? 1–10	
How bothered are you by Adjacent patient noise (music or tv)? 1–10	
How bothered are you by the sound of Procedure noise? 1–10	
Does noise in the SICU affect your level of tiredness? 1–10	
Has noise ever interfered with your willingness to sign a consent form for a procedure? 1–10	Question 9: Has noise ever interfered with your willingness to sign a consent form for a procedure? 1–10
How much does tiredness impact pain of minor procedures? 1–10	Question 10: How much do you think noise impacts the pain of minor procedures (IV lines and blood draws) 1–10/Open text
How much does tiredness impact the pain of the following major procedures:	
Central Lines 1–10	Question 11: How much do you think noise impacts pain of the following major procedures: Central lines, Chest tubes, Procedure involving the staff sterile prepping and draping? 1–10/Open text
Chest tubes 1–10	
Procedures involving the staff (sterile prepping and draping) 1–10	
Were you taught any relaxation techniques before your SICU stay? If yes, which technique? Open text	Question 12: Were you taught any relaxation techniques before you SICU stay? Yes-no/open text
	Question 13: Were you taught any relaxation techniques during your SICU stay? Yes-no/open text
Have you or family members developed any relaxation technique on your own during your SICU stay? If yes, name of technique; Open text	Question 14: Have you or your family members developed any relaxation techniques on your own during your SICU stay? Yes-no/open text
What are your ideas for noise reduction and improved relaxation in the SICU? Open text	Question 15: What are your ideas for noise reduction and improved relaxation in the SICU? Open text
STAFF	
What percent of patients complain about the noise in the SICU? 0–100%	Do patients seem more restless or combative when the SICU is noisy? 0–10 (never–always)
What percent of family members complain about the noise in the SICU? 0–100%	Do you notice any influences upon treatment due to noise? 0–10 (never–always)
How often do patients and families request attempts at noise reduction? 0–10 (never–endlessly)	Is there any increased refusal to sign consents when the unit is noisy? 0–10 (never–always)
Please rate the following according to levels of complaints 0–10 (never-sometimes-often-always)	Do you think noise reduction affects outcome in the SICU? 0–10 (never–always)
Vents IVACS	Do you think it affects patient and family interaction with staff? 0–10 (never–always)
Monitor Alarms Voice Noise	Do you think noise reduction affects outcome in the SICU? 0–10 (never–always)
Adjacent patient noise Procedure noise	Do you think noise affects patient and family interaction with staff? 0–10 (never–always)
